# AXL in cancer: a modulator of drug resistance and therapeutic target

**DOI:** 10.1186/s13046-023-02726-w

**Published:** 2023-06-16

**Authors:** Yaoxiang Tang, Hongjing Zang, Qiuyuan Wen, Songqing Fan

**Affiliations:** Department of Pathology, The Second Xiangya Hospital, Central South University, Changsha, Hunan 410011 China

**Keywords:** AXL, Cancer, Drug resistance, Target therapy, Molecular mechanisms

## Abstract

AXL is a member of the TAM (TYRO3, AXL, and MERTK) receptor tyrosine kinases family (RTKs), and its abnormal expression has been linked to clinicopathological features and poor prognosis of cancer patients. There is mounting evidence supporting AXL's role in the occurrence and progression of cancer, as well as drug resistance and treatment tolerance. Recent studies revealed that reducing AXL expression can weaken cancer cells' drug resistance, indicating that AXL may be a promising target for anti-cancer drug treatment. This review aims to summarize the AXL's structure, the mechanisms regulating and activating it, and its expression pattern, especially in drug-resistant cancers. Additionally, we will discuss the diverse functions of AXL in mediating cancer drug resistance and the potential of AXL inhibitors in cancer treatment.

## Background

Cancer remains a leading cause of death worldwide, with its incidence and mortality burden continuing to rise rapidly [1]. Despite significant progress in research and the development of cancer treatment strategies such as targeted therapy and immunotherapy, drug resistance remains a major obstacle to effective therapeutic interventions against cancer [2, 3]. The drug resistance can be intrinsic or acquired and reflects the result of multiple genetic and epigenetic alterations in cancer cells [4, 5]. Therefore, urgent measures must be taken to identify new therapeutic strategies that can target intrinsic and acquired resistance mechanisms.

AXL is a member of the TAM (TYRO3, AXL, and MERTK) receptor tyrosine kinases (RTKs) family [6]. By binding to its primary ligand, the growth arrest-specific protein 6 (GAS6), AXL participates in various signal transduction cascades and plays a critical role in various biological processes including cell proliferation, survival, migration, efferocytosis, angiogenesis, platelet aggregation and fibrosis, and regulation of natural killer (NK) cell development [7, 8]. Multiple lines of evidence indicate that AXL is also involved in cancer progression and treatment tolerance. For example, in lung cancers, AXL interacts with epidermal growth factor receptor (EGFR) and human epidermal growth factor receptor 3 (ERBB3, also known as HER3) to maintain the activation status of downstream signal pathway, which confers intrinsic resistance to osimertinib in non-small cell lung cancer (NSCLC) cells [9]. AXL also promotes the transcription level of MYC, which leads to the imbalance of purine metabolism and accelerates the emergence of drug-resistant mutations in NSCLC [10]. In colorectal cancers, AXL induces the expression of Twist family BHLH transcription factor 1 (TWIST1) and mediates resistance to polo-like kinase 1 (PLK1) inhibitor [11]. Overexpression of AXL has also been found in a variety of other cancers, including clear cell renal cell carcinoma (ccRCC) [12], hepatocellular carcinoma (HCC) [13], and cholangiocarcinoma (CCA) [14], which is associated with poor prognosis of these cancer patients [12–14]. These findings suggest that AXL could be a useful therapeutic target to address the issue of cancer drug resistance.

This review aims to describe the structure of AXL and its expression regulation, summarize the expression pattern of AXL in cancers, and further discuss the role of AXL plays in cancer occurrence development, particularly in anti-cancer drug resistance. Additionally, we will explore the potential of AXL as a therapeutic target to overcome tumor chemoresistance.

## Structure and activation of AXL

**Fig. 1:**
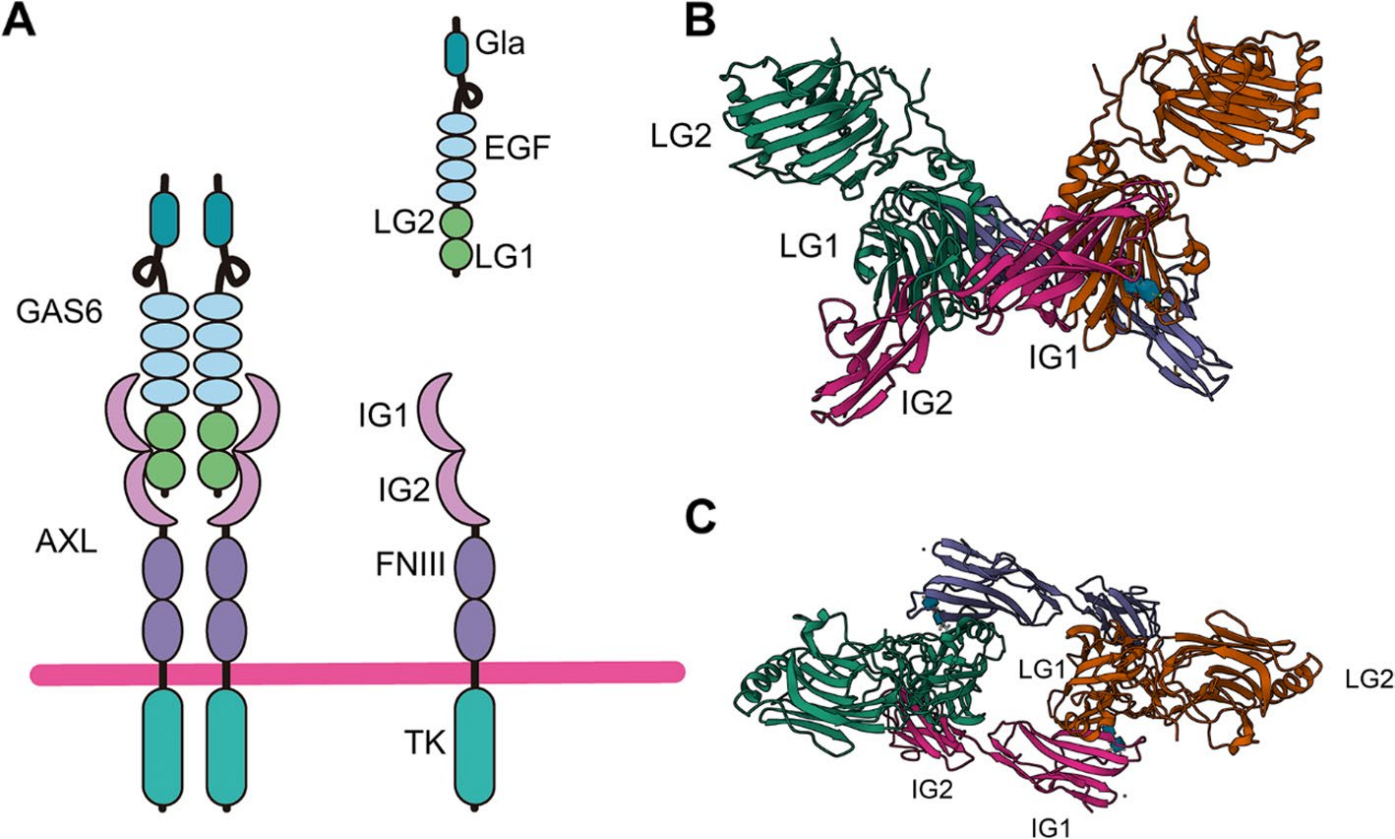
The structure of AXL and GAS6. **A** The AXL protein comprises an intracellular domain, single helix transmembrane region, two fibronectin type III (FNIII) domains, and two immunoglobulin (IG)-like domains. On the other hand, GAS6 consists of a γ-carboxyglutamic acid (GLA) domain, a loop region, four epidermal growth factor (EGF)-like repeats, and two globular laminin G like (LG) domains. In Figure **B** and **C**, we can observe the interaction between AXL and GAS6, front both the front and top sides respectively, as visualized in the Protein Data Bank (PDB) with the identifier 2C5D

The AXL gene is located on chromosome 19q13.2, it includes 20 exons and encodes an 894-amino acid polypeptide with multiple domains, which can be divided into three parts (Fig. 1A) [15]. Exons 1–10 mainly encode two fibronectin type III (FNIII) domains and two immunoglobulin (IG)-like domains constituting the extracellular part, which is involved in binding with ligands (Fig. 1B and C). Exon 11 encodes extracellular proteolytic cleavage sites and a transmembrane domain, and exons 12–20 encode intracellular domain with tyrosine kinase activity [15, 16].

The main ligand of AXL [17] is GAS6, which consists of a γ-carboxyglutamic acid (GLA) domain at N-terminus, a loop region, four EGF-like repeats in the middle, and two globular laminin G like (LG) domains at C-terminus [18]. When AXL binds to GAS6, the complex dimerizes with another GAS6-AXL complex to form a 2:2 homodimerized complex with no direct AXL/AXL or GAS6/GAS6 contact, followed by trans-autophosphorylation of the tyrosine residues in the intracellular domain of AXL [19–21]. AXL phosphorylation is required for recruitment of corresponding adaptor molecules and effector proteins and ultimately to activate downstream signal pathways [19–21]. Six phosphorylation sites have been found in the AXL kinase domain, of which three (Tyr698, Tyr702 and Tyr703) are considered to be related to autophosphorylation and AXL activation, while the other three (Tyr779, Tyr821 and Tyr866) are involved in the docking and signal transduction of adaptor proteins [22].

In addition to GAS6, protein S (PROS1) has also been identified as a ligand of AXL. By phosphorylating AXL and activating downstream NF-κB signal pathway, PROS1 promotes Glioblastoma (GBM) tumor growth [23]. Besides AXL’s ligands, other TAM family members [24] or non-TAM proteins also affect AXL activation. For instance, co-immunoprecipitation experiments suggest that the AXL and TYRO3 receptors are closely associated, which significantly enhance GAS6 mediated AXL phosphorylation [24, 25].

Furthermore, AXL can be activated by interacting with several other non-TAM family member proteins via GAS6 independent mechanisms. For instance, it has been found that AXL heterodimerize with ERBB receptor family members, platelet-derived growth factor receptor (PDGFR), and mesenchymal to epithelial transition factor (MET) and is activated without GAS6 participation [26–28]. In addition, activated EGFR phosphorylates AXL Tyr779, leading to ligand-independent AXL activity and activation of more diversified down-stream signaling pathways than those triggered by EGFR alone [26, 29]. Similarly, the activated HER2 forms a complex with AXL and activates AXL in a GAS6-independent manner, which accelerates epithelial-mesenchymal transition (EMT) and metastasis of breast cancer cells [30]. AXL is required for vascular endothelial growth factor-A (VEGF-A) dependent activation of PI3K (phosphoinositide 3-kinase)/AKT (protein kinase B) in endothelial cells. Under the stimulation of VEGF-A, VEGF receptor-2 (VEGFR-2) activates the Src family kinase (SFK), and then promotes the GAS6-independent activation of AXL, which is necessary for vascular permeability and corneal neovascularization [31].

## Regulation of AXL

**Fig. 2:**
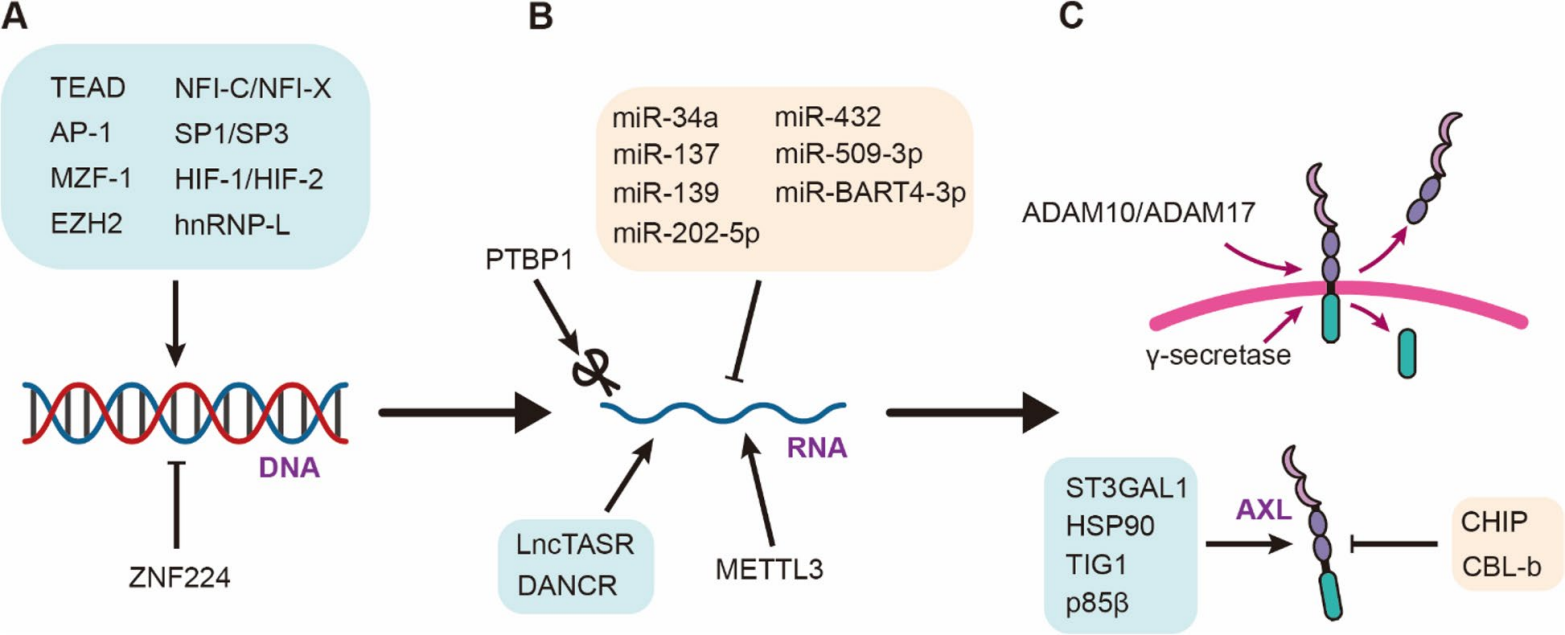
The regulation of AXL. **A** Expression of AXL is regulated by various transcription factors. **B** AXL undergoes post-transcriptional regulation. **C** The protein level of AXL is regulated in the post-translation stage

In recent years, AXL has emerged as a key player in various biological processes, including immune regulation, cellular signaling, and cancer progression [7, 8]. Consequently, the regulation of AXL has become an area of intense research interest. Several mechanisms have been identified that regulate AXL expression and function, including transcriptional, post-transcriptional, and post-translational regulation (Fig. 2).

Transcriptional regulation of AXL is achieved through the binding of various transcription factors to the AXL promoter region, such as TEA domain (TEAD), activation protein-1 (AP-1), and hypoxia-inducible transcription factor-1α (HIF-1α) [32–34]. TEAD combines with AXL promoter to enhance its promoter activity, in turn mediating the resistance of colorectal cancer to 5-FU [34]. AP-1 can bind to AP-1 motif of Axl promoter [32]. Blocking JNK (c-Jun N-terminal kinase)/AP-1 inhibits AXL transcription and attenuates drug resistance to PI3Kα therapy in esophagus cancer and head and neck squamous cell carcinoma [35]. Additionally, epigenetic modifications have been shown to modulate AXL expression. Some studies have found that methylation in the GC rich region of AXL promoter can restrict AXL gene expression [36].

Post-transcriptional regulation of AXL involves non-coding RNAs. Various micro-RNAs (miRNAs), such as miR-34a, miR-432, and miR-202-5p, can negatively regulate AXL expression by binding to the 3'UTR of the AXL mRNA and promoting its degradation [37–39]. LncTASR, a long noncoding RNA (lncRNA), can directly bind to 5′ UTR of AXL mRNA to stabilize AXL mRNA [40]. Additionally, alternative splicing of the AXL transcript can result in the production of different AXL isoforms, which may have distinct functions and regulation [41].

Finally, post-translational regulation plays a critical role in maintaining protein stability and activity. For instance, by mediating AXL sialylation, ST3 β-Galactoside α-2,3-Sialyltransferase 1 (ST3GAL1) can increase the affinity between AXL and GAS6, and induces activation of AXL [42]. Heat shock protein 90 (HSP90), a molecular chaperone, can correctly fold proteins and be required to stabilize AXL [43], while other molecules, such as carboxy terminus of HSP70 interacting protein (CHIP) and E3 ubiquitin ligase CBL-b (casitas B lymphoma-b), can ubiquitinate AXL protein and induce its proteasome degradation [44–46]. Additionally, AXL can be cleaved by the A disintegrin and metalloproteinases (ADAM) 10 and ADAM17 to generate soluble AXL (sAXL), which inhibit AXL function and could be a promising biomarker for predicting cancer progression [47].

In summary, the regulation of AXL is a complex process that involves various mechanisms, including transcriptional, post-transcriptional, and post-translational regulation. A better understanding of these mechanisms is crucial for the development of novel therapeutic strategies targeting AXL in cancer and other diseases.

## Expression of AXL in cancers

The receptor tyrosine kinase AXL has been shown to be highly expressed in several major types of cancers and is closely associated with tumor progression [48]. In particular, upregulated AXL mRNA expression has been observed in ccRCC, where it is associated with worse overall survival (OS) and can serve as an independent predictor of prognosis in ccRCC patients [12]. Moreover, high AXL expression has been reported in CCA compared to the adjacent normal tissue, the patients with high AXL levels have a higher risk of developing metastasis and a shorter OS [14]. Similarly, in HCC patients, high AXL expression could serve as a biomarker of higher recurrence and lower OS after hepatectomy [13]. Notably, higher AXL expression is a potent independent predictor of poor progression-free survival (PFS) or OS in patients with HPV-negative tumors treated by surgery alone [49] and patients with lung adenocarcinoma [50].

Furthermore, increased AXL mRNA and/or protein has been observed in other cancers, such as papillary thyroid carcinoma [51] and pancreatic ductal adenocarcinoma (PDAC) [52]. The enzymatic processing of AXL leads to the production of sAXL, and plasma sAXL is significantly increased in HCC and PDAC, making it a candidate biomarker for early diagnosis of these cancers [52–55], thus highlighting the potential value of sAXL in cancer diagnosis and prognosis prediction.

In recent years, studies have shown that high AXL expression is closely associated with treatment-resistant cancers (Table 1). For instance, high AXL expression has been found in NSCLC patients with low response to EGFR tyrosine kinase inhibitor (TKI) [9], as well as in erlotinib or osimertinib resistant cancer cell lines [56, 57]. Similarly, in patients with ovarian cancer or endometrial cancer, high AXL expression is associated with poor chemoresponse [58, 59] and patients with ccRCC who have high AXL expression shows lower objective response rate to PD-1 inhibition therapy [60]. In patients with colorectal cancer who received anti-EGFR treatments, those with high AXL expression show lower PFS rates than those with low AXL [61]. In addition, cell line expression data also reveal that high AXL expression is found in drug-resistant breast cancer cells and small-cell lung cancer cells (SCLC), but not in drug-sensitive cells [62, 63]. Overall, these findings suggest that AXL may play a critical role in promoting resistance to cancer treatments and further highlight the potential of AXL as a therapeutic target for cancer treatment.

**Table 1:** The expression of AXL in drug-resistant cancers

Type of resistance	Drug	Cancer type	Model	Significance	Reference
**Chemotherapy**	Paclitaxel, carboplatin	Ovarian cancer	Human	AXL expression was higher in tumors with a poor response to chemotherapy	[59]
	Paclitaxel	Endometrial Cancer	Human	AXL level was lower in tumors with good chemoresponse than in those with a poor response	[58]
**Targeted therapy**	Osimertinib	NSCLC	Human	The response rate of patients with low AXL expression for osimertinib was higher than that with high AXL expression	[19]
			Cell	expression of AXL protein was also higher in osimertinib-resistant cells (H1975 OR1 and H1975 OR2) than sensitive cell lines (H1975)	[56]
	Erlotinib	NSCLC	Human	AXL significantly increased in NSCLC patients with erlotinib resistance	[57]
			Cell	Compared with sensitive cell lines (HCC827 and HCC4006), the mRNA and protein of AXL in erlotinib-resistant cells (HCC8267 ER and HCC4006 ER) were significantly up-regulated	
	Cetuximab/panitumumab	Colorectal cancer	Human	Progression-free survival was significantly lower in RAS-WT patients with high-AXL undergoing anti-EGFR therapy	[61]
	Trastuzumab	Breast cancer	Cell	AXL was up-regulated in resistant cell lines (AU565R, BT474R, and SKBR3R) compared to corresponding sensitive cell lines (AU565, BT474, and SKBR3)	[62]
			Mouse	AXL mRNA level is significantly higher in vivo PDX–resistant model	
			Human	Patients who later experienced tumor recurrence have higher expression of AXL in the initial diagnosis	
	AZD1775	Small Cell Lung Cancer	Cell	Compared with sensitive cell lines (H1836, H82, H1048), the AXL expression of resistant cell lines (H1417, H865, H1930) was significantly higher	[63]
**Immunotherapy**	Nivolumab	ccRCC	Human	The objective response rate was significantly lower in patients with high AXL level. Survival rate of patients with AXL-high and PD-1-positive undergoing PD-1 block therapy was significantly lower	[60]

## Functions of AXL in drug-resistant cancer

**Fig. 3:**
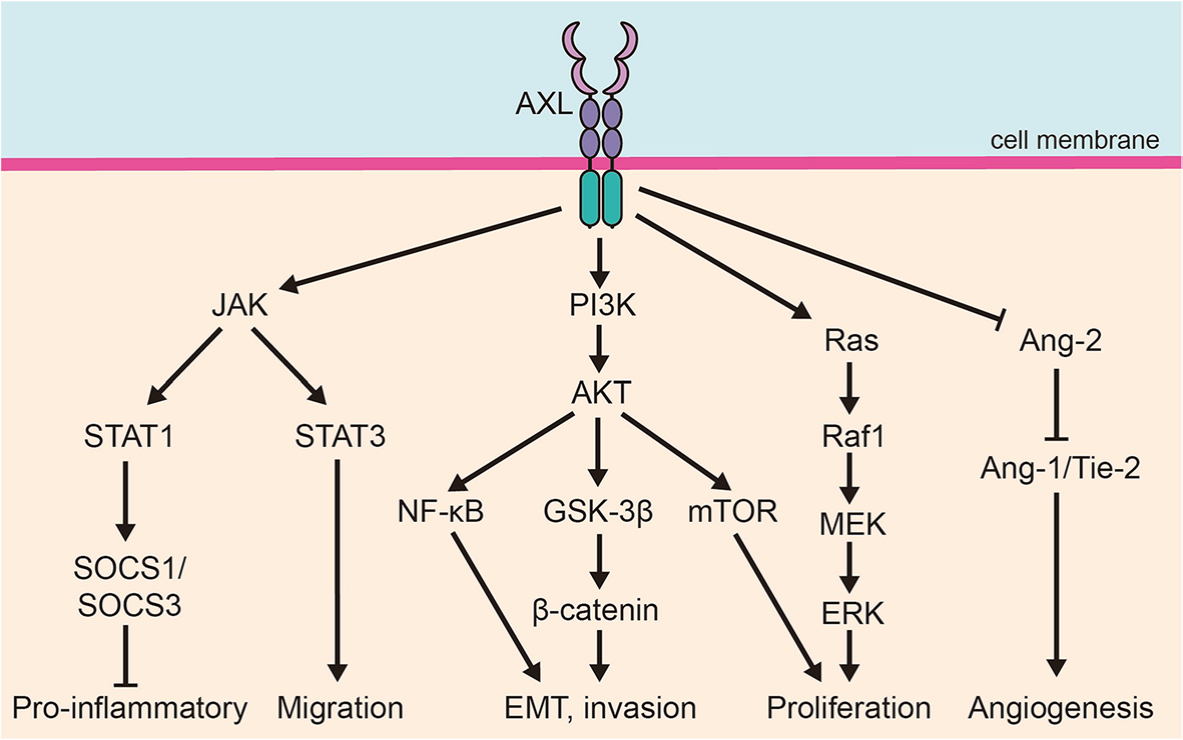
Signal pathways mediated by AXL in the occurrence and development of cancer. AXL play a crucial role in the occurrence and development of cancer through various signal pathways

Multiple studies have demonstrated that AXL is involved in various signaling pathways that are critical for cancer initiation and progression (Fig. 3). However, the precise mechanisms underlying AXL-mediated drug resistance in cancer cells remain largely unclear. Here, we will specifically discuss the role AXL in promoting drug tolerance of cancer cells to treatment.

### EMT

**Fig. 4:**
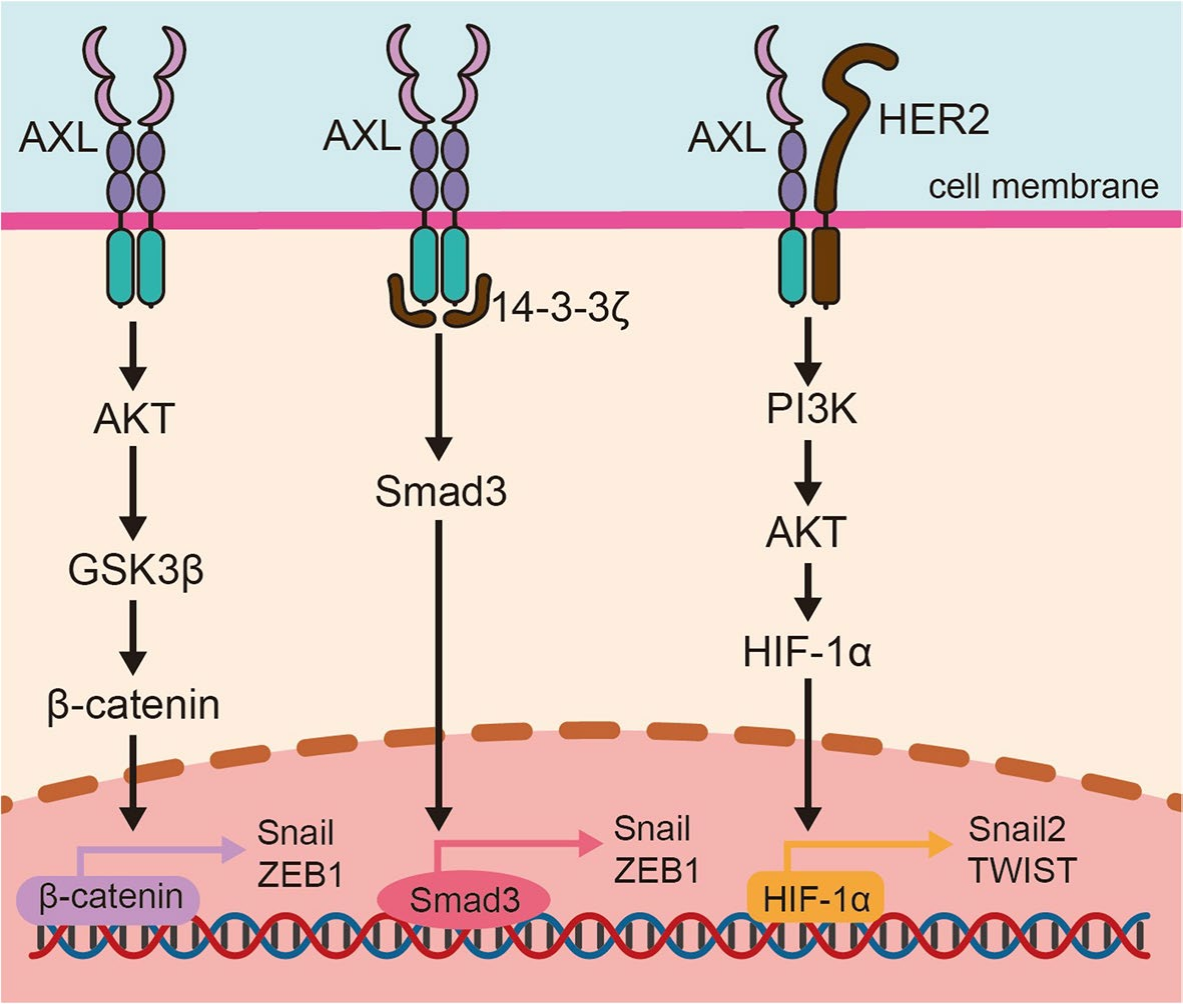
AXL-mediated epithelial–mesenchymal transition (EMT) in drug-resistant cancer. AXL is known to activate EMT transcription factors through three pathways to promote EMT transformation and drug resistance of cancer cells. These pathways include AKT/GSK-3β/β-catenin, TGF-β/Smad3, and PI3K/AKT/HIF-1α

EMT is a critical process by which epithelial cells acquire a mesenchymal phenotype, which enables them to invade and metastasize to distant organs. This mechanism has been shown to contribute to drug resistance in cancer cells [64–66]. A growing body of evidence suggests that AXL is closely associated with EMT (Fig. 4), and can be used as an EMT marker in several types of cancer, including esophageal squamous cell carcinoma (ESCC) [67]. In oral squamous cell carcinoma (OSCC), AXL upregulates Snail expression and promotes EMT via AKT/GSK-3β (Glycogen Synthase Kinase 3β)/β-catenin signaling pathway [68]. AXL also induces the upregulation of ZEB1 (zinc finger E-box binding homeobox 1) transcription and mediates the drug resistance of breast cancer to doxorubicin through the same pathway [69]. In colorectal cancer, increased AXL contributes to the upregulation of TWIST1, which is directly related to EMT and mediates resistance to PLK1 inhibitors [11]. Previous studies have confirmed that TGF-β (transforming growth factor-β)/SMAD3 (SMAD family member 3) participates in EMT of lung adenocarcinoma cells [70]. In HCC, the interaction between AXL and 14–3-3ζ leads to phosphorylation of Ser213 in SMAD3, inducing the upregulation of TGF-β target genes such as Snail and autocrine TGF-β secretion [71].

On the other hand, inhibition of GAS6/AXL axis reduces Snail and N-cadherin but upregulates E-cadherin, inhibiting EMT in esophageal cancer cells [72]. Similarly, miR-625-3p can directly target AXL and reverse TGF-β1 induced EMT, enhancing sensitivity to gefitinib in NSCLC [73]. Hypoxia has been shown to increased activity of HIFs and promote tumor progression by inducing EMT [74]. Interference with AXL leads to downregulation of HIF-1α, which, in turn, reduces EMT induced by hypoxia and enhances the immunotherapeutic responses in HER2 breast cancer [75].

### DNA damage and DNA damage response (DDR)

**Fig. 5:**
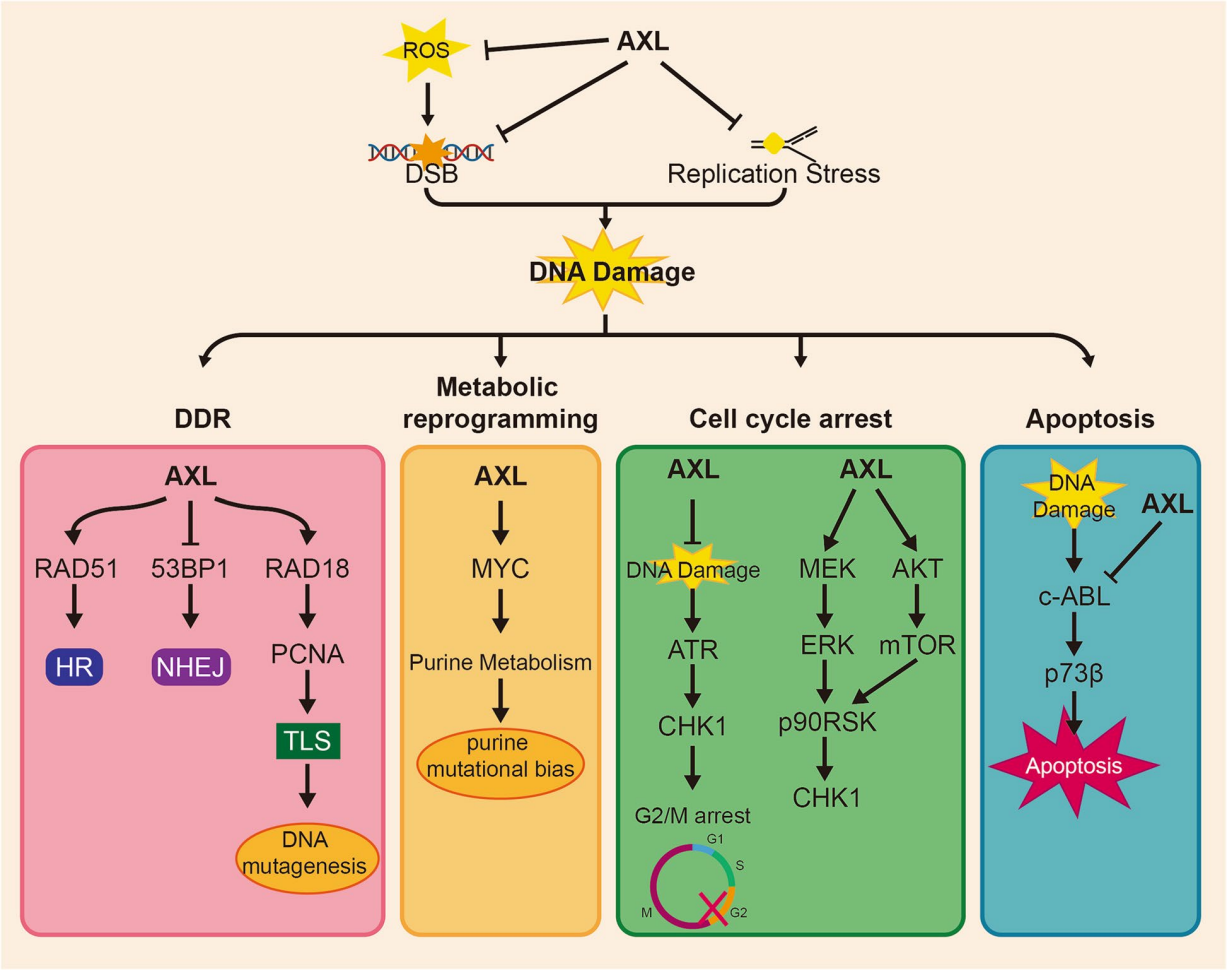
AXL affect DNA damage and DNA damage response (DDR) in drug-resistant cancer. DNA damage and repair are dynamic processes that require a delicate balance. AXL plays a crucial role in maintaining this balance by not only inhibiting DNA damage but also participating in multiple processes of DNA repair, such as DNA damage response (DDR), metabolic reprogramming, cell cycle arrest, and apoptosis

Stimulation such as ultraviolet light can cause DNA replication stress (RS), leading to stalled replication forks and DNA damage that introduces genomic instability [76]. Genomic instability is a key hallmark of cancer and is closely linked to drug-resistance [77]. Cancer cells respond to DNA damage through various ways, collectively known as DDR (Fig. 5). These responses can be summarized into four aspects: activation of various repair pathways, metabolic reprogramming, blocking cell cycle process, and inducing cell death in the cases of irreparable damage [78–80]. It has been well established that aberrant DDR contributes to cancer progression and resistance to DNA-damaging drugs.

Several studies have revealed that AXL is associated with DNA damage. AXL has been found to attenuate reactive oxygen species (ROS) production and inhibit DNA double-strand breaks (DSB) [10], on the other hand, inhibition of AXL leads to enhanced RS and increased DNA damage [59]. Additionally, when combined with other drugs, AXL can interfere with replication fork progression. Although carboplatin and GAS6 inhibitor AVB500 alone shows no effect on replication forks, the combination of the two significantly hinder the progress of replication forks in ovarian cancer cells [59]. Similarly, use of AXL inhibitor BGB324 with ATR inhibitors can cause collapse of replication fork in NSCLC [81]. It is worth noting that the combination of PARP inhibitor and AVB-500 increases DNA damage and genomic instability by increasing replication fork speed rather than stalling the fork. In addition, to affecting DNA replication process, AXL is also involved in multiple repair pathways of DDR [59]. Inhibition of GAS6/AXL axis reduces RAD51 foci and increase 53BP1 foci, inhibiting homologous recombination (HR) and increasing the sensitivity of ovarian cancer to carboplatin [59]. Translesion synthesis (TLS) is a basic pathway for repairing DNA damage caused by replication arrest, but it also serves as the main source of cell mutation [82]. Mono-ubiquitination of proliferating cell nuclear antigen (PCNA) induced by RAD18 is critical for TLS [83]. A recent study found that AXL neddylates and activates RAD18 to enhance TLS and accelerate the emergence of T790M in resistant NSCLC cells [10].

Furthermore, AXL is involved in upregulation and activation of MYC, leading to an imbalance in purine metabolism and increased adaptive mutability [10]. C-ABL and p73 play important roles in the process of cellular apoptosis caused by DNA damage [84], and both are interfered with by AXL to enhance cisplatin resistance in esophageal cancer [85] through impeding nuclear aggregation of c-ABL and impairing p73 protein stability. The effect of AXL on cell cycle progression is still under debate. One study found that AXL inhibition activates ATR/CHK1 (checkpoint kinase 1) and sensitizes NSCLC cells to ATR inhibitors. The combination of AXL and ATR inhibitors can prematurely activate cyclin dependent kinase 1 (CDC2) and induce mitotic catastrophes [81]. However, other have reported AXL overexpression activates the ERK/p90RSK and mTOR pathways, thereby activating CHK1 to promote cell survival and mediate primary and acquired resistance to WEE1 inhibition in SCLC [63]. In summary, AXL has been demonstrated to mediate drug resistance by influencing DNA damage and repair, but its intrinsic mechanism for DDR remains to be specifically explored.

### Immunosuppression

**Fig. 6:**
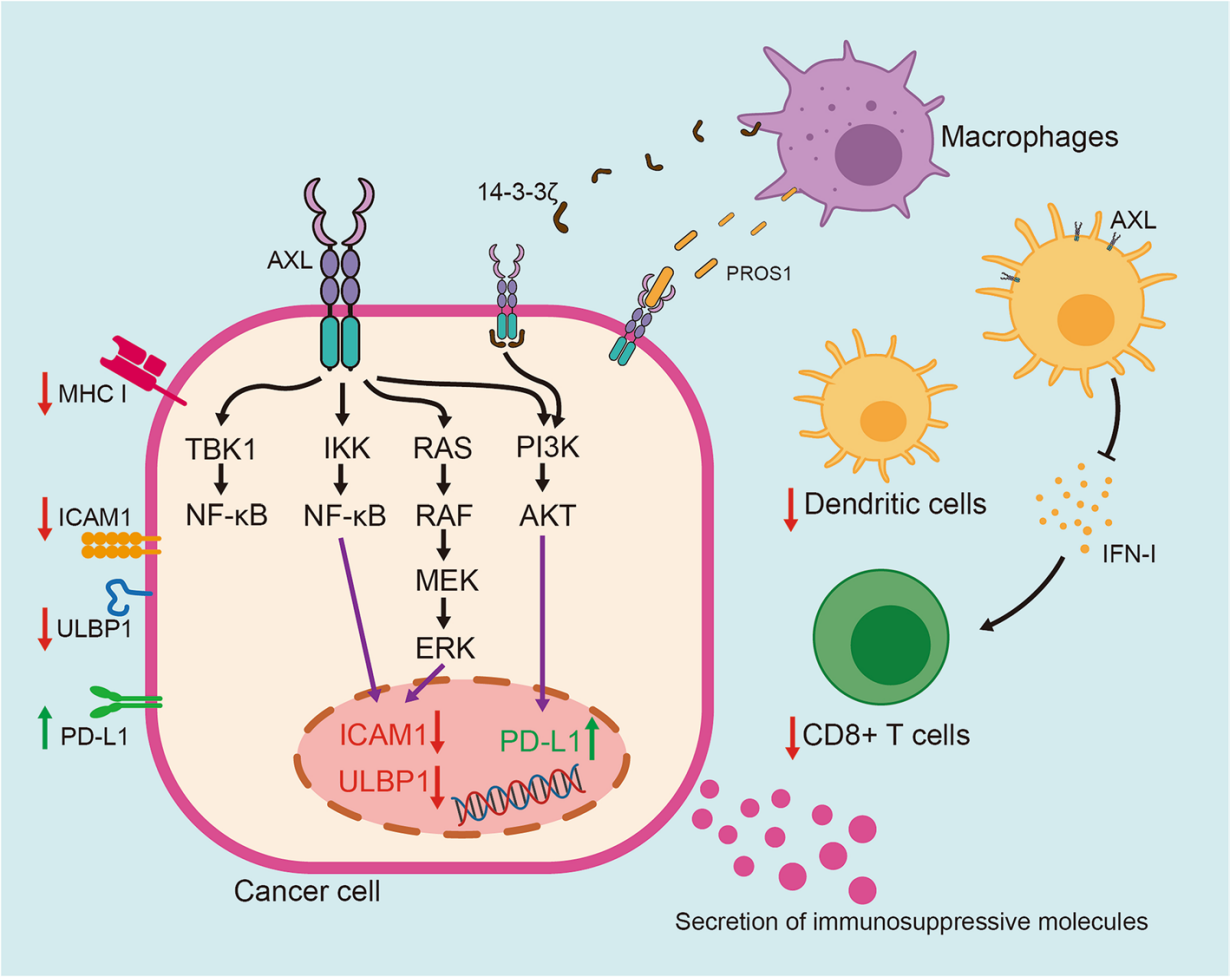
AXL-mediated immunosuppression in drug-resistant cancer. AXL plays a key role in mediating immunosuppression through both intrinsic shaping of cancer cells and extrinsic modification of microenvironment surrounding tumors

Although significant progress has been made in immunotherapy by activating the immune system to eliminate tumors in recent years, drug resistance remains a major obstacle to its application in clinical practice [86]. AXL plays an essential role in shaping the process of tumor immune tolerance (Fig. 6) [87]. AXL endows cancer cells with resistance to treatment through activation of PI3K/AKT pathway and up-regulation of programmed cell death ligand 1 (PD-L1) transcription in head and neck cancer cells, thereby inhibiting the immune killing effect of the body [88].

In the case of GBM, immune-related cells in tumor microenvironment, such as tumor- associated microglia and macrophages, secrete PROS1 to promote the growth of glioma cells, which phosphorylates and activates AXL in glioma stem cells, thus inhibition of AXL could significantly improve the efficacy of Navurizumab [23]. Additionally, tumor-related macrophages secrete 14–3-3ζ, which interacts and phosphorylates AXL to activate downstream pathways to promote the tolerance of PDAC cells to chemotherapy [89]. AXL could affect cytotoxic immune response against tumors by reducing the expression of intercellular adhesion molecule 1 (ICAM1) and UL16 binding protein 1 (ULBP1) in mesenchymal human lung cancer cells. Both of these contribute to the immune resistance to NK and cytotoxic T lymphocytes (CTL) cells [90]. Since ULBP1 plays a role in the recognition of target cells [91] and the combination of ICAM1 and lymphocyte function-associated antigen 1 (LFA-1) enhances immune cells to kill the target cells [92], targeting AXL is beneficial to the formation of anti-tumor microenvironment and enhances the treatment response. Indeed, depletion of AXL facilitates expression of MHC-I, infiltration of dendritic cells and CD8 + T cells, and T-cell-mediated immune response [93]. Particularly, inhibiting AXL in dendritic cells can induce the secretion of type I interferon, promote the expansion of CD8 + T cells, and sensitize NSCLC carrying serine/threonine kinase 11 (STK11/LKB1) mutant to pabolizumab [94]. Recent study in leukemia cells has found GAS6/AXL axis is required to skew macrophages toward a tumor-promoting tissue repair phenotype to establish a suppressive environment to prevent immune attacks [95]. It has been proposed that AXL inhibition can be achieved by blocking TBK1 (TANK binding kinase 1)/NF-κB pathway, a key signal pathway of immune cells, changes the composition of chemokines and cytokines in tumor microenvironment, making tumor sensitive to treatment [96].

### Activation of oncogenic bypass pathway

Ample evidence suggests AXL mediates resistance to multiple anti-cancer drugs. In NSCLC with EGFR mutation, the activation of several signal pathways, such as MAPK/ERK and PI3K/AKT, is considered to be one of the major mechanisms responsible for acquiring drug resistance against EGFR TKIs [97]. In the case of resistance to osimertinib, multiple pathways seem to be involved, including the MAPK/ERK pathway, SRC and its downstream AKT signal pathway, and EGFR-induced signal transduction triggered by AXL [9, 56, 98–100]. In breast cancers, the heterodimerization of AXL and HER2 leads to the acquired resistance to anti-HER2 drug trastuzumab by activating AKT and ERK pathways [62].

### Other functions

AXL can contribute to drug tolerance, and its inhibition has been shown to reduce drug resistance in several types of cancer. For instance, inhibition of AXL reduces the phosphorylation of M2 isoform of pyruvate kinase (PKM2) at Y105, which decreases glycolysis and enhances chemosensitivity of human ovarian cancer cells to cisplatin [101]. In endometrial cancer cells, inhibition of AXL down-regulates various glycolytic metabolites, leading to increased sensitivity to paclitaxel [58]. AXL has also been found to activate Akt/β-catenin pathway which up-regulates the transcription level of c-MYC and promotes resistance of esophageal adenocarcinoma to epirubicin [102]. Furthermore, AXL can mediate the resistance of head and neck cancer to cetuximab through two mechanisms: (1) Activation of HER3 via up-regulation of HER3 ligand neuromodulator 1 (NRG1) [103]; and (2) Activation of c-ABL kinase via Tyr821 of AXL [104].

## Targeting AXL to surmount drug resistance in cancer therapy

There is ample evidence supporting the important role of AXL in drug resistance across various types of cancers. Therefore, targeting AXL is a promising strategy for addressing drug resistance. Drugs that inhibit AXL can be grouped based on their mechanisms of action, including small molecule selective inhibitors (such as BGB324 and TP-0903), antibody–drug conjugates (such as BA3011), anti-AXL Fc fusion protein AVB-S6-500, and multitargeted inhibitors (such as ONO-7475, Merestinib and Sitravatinib) [105]. Experimental data from both in vivo and in vitro studies suggest that carboplatin/paclitaxel combined with AVB-S6-500 is more effective than chemotherapy alone [59]. Additionally, the combination of nivolumab and BGB324 prolongs the survival period of mice with GBM [23], and the combination of TP0903 and WEE1 inhibitor can overcome the resistance of SCLC to WEE1 inhibitor [63]. Table 2 listed AXL inhibitors that have entered clinical trials, some of which may help to overcome drug resistance and enhance treatment sensitivity when combined with other therapies.

**Table 2:** AXL-targeted drugs in clinical trials

Drug	Cancer	Combination with	Clinical Trial No	Phase
**Bemcentinib (BGB324, R428)**	Advanced Adenocarcinoma of the Lung	Pembrolizumab	NCT03184571	Phase 2
	Triple Negative Breast Cancer	Pembrolizumab	NCT03184558	Phase 2
	Stage IIIb or Stage IV non-small cell lung cancer (NSCLC)	Erlotinib	NCT02424617	Phase 1/2
	Advanced or metastatic non-squamous NSCLC	Pembrolizumab/ Pemetrexed/ Carboplatin	NCT05469178	Phase 1/2
	Acute Myeloid Leukemia (AML)	Cytarabine/ Decitabine	NCT02488408	Phase 1/2
**Dubermatinib (TP-0903)**	FLT3 gene mutated AML	Azacitidine	NCT04518345	Phase 1/2
**DS-1205c**	Metastatic or Unresectable Epidermal Growth Factor Receptor (EGFR)-Mutant NSCLC	Osimertinib	NCT03255083	Phase 1
	Metastatic or Unresectable EGFR-Mutant NSCLC	Gefitinib	NCT03599518	Phase 1
**BA3011 (CAB-AXL-ADC)**	Metastatic NSCLC	PD-1 inhibitor	NCT04681131	Phase 2
	Solid tumors	PD-1 inhibitor	NCT03425279	Phase 1/2
**AVB-S6-500 (batiraxcept)**	Advanced Urothelial Carcinoma	Avelumab	NCT04004442	Phase 1
	Advanced Pancreatic Adenocarcinoma	Nab paclitaxel/ Gemcitabine	NCT04983407	Phase 1/2
	Platinum-Resistant Recurrent Ovarian Cancer (OC)	Paclitaxel/ Pegylated liposomal doxorubicin	NCT03639246	Phase 1
	Platinum-Resistant Recurrent OC	Paclitaxel	NCT04729608	Phase 3
	Platinum-Resistant or Recurrent Ovarian, Fallopian Tube, or Primary Peritoneal Cancer	Durvalumab	NCT04019288	Phase 1/2
	Advanced or Metastatic Clear Cell Renal Cell Carcinoma (ccRCC)	Cabozantinib/ Nivolumab	NCT04300140	Phase 1/2
**ONO-7475**	Acute Leukemias	Venetoclax	NCT03176277	Phase 1/2
**Merestinib (LY2801653)**	Relapsed or Refractory AML	LY2874455	NCT03125239	Phase 1
	Advanced or Metastatic Cancer	Cisplatin/ Gemcitabine	NCT03027284	Phase 1
	Advanced Refractory Solid Tumors	LY3300054	NCT02791334	Phase 1
	Advanced Cancers	Ramucirumab (LY3009806)	NCT02745769	Phase 1
	Advanced or Metastatic Biliary Tract Cancer	cisplatin and gemcitabine	NCT02711553	Phase 2
**Sitravatinib (MGCD516)**	Recurrent/Metastatic Cervical Cancer After Platinum-Based Chemotherapy	Tislelizumab	NCT05614453	Phase 2
	hepatocellular carcinoma (HCC) at high risk of recurrence after curative resection	Tislelizumab	NCT05407519	Phase 2
	HCC at high risk of recurrence after curative resection	Tislelizumab	NCT05564338	Phase 3
	ccRCC	Nivolumab	NCT03680521	Phase 2
	Recurrent Endometrial Cancer and Other Solid Tumors with Deficient Mismatch Repair System	Pembrolizumab	NCT05419817	Phase 2
	Advanced Non-Squamous NSCLC	Nivolumab	NCT03906071	Phase 3
	Metastatic or Advanced ccRCC	Nivolumab	NCT04904302	Phase 2
	Advanced Treatment-Naïve PD-L1 + Non-Squamous NSCLC	Pembrolizumab	NCT04925986	Phase 2
	Advanced, Unresectable NSCLC	Tislelizumab	NCT05176925	Phase 2
	Extensive stage small cell lung cancer	Tislelizumab	NCT05228496	Phase 2
	Advanced or Metastatic NSCLC	Tislelizumab	NCT04921358	Phase 3
	Unresectable or Metastatic Melanoma	Tislelizumab	NCT05104801	Phase 2
	Esophageal Squamous Cell Carcinoma	Tislelizumab	NCT05461794	Phase 2
	Recurrent or Metastatic TNBC	Tislelizumab	NCT04734262	Phase 2
	Metastatic Uveal Melanoma with Liver Metastases	Tislelizumab	NCT05542342	Phase 2
	NSCLC	Nivolumab	NCT02954991	Phase 2
	Urothelial Carcinoma	Nivolumab/ Pembrolizumab/ Enfortumab vedotin	NCT03606174	Phase 2
	Advanced Solid Tumors	Tislelizumab	NCT03666143	Phase 1
	Advanced or Metastatic HCC or Gastric/Gastroesophageal Junction Cancer (GC/GEJC)	Tislelizumab	NCT03941873	Phase 1/2

In addition to the AXL-targeted drugs mentioned above, many monoclonal antibodies specific to AXL have been shown to inhibit the growth of cancer cells, including YW327.6S2, 20G7-D9, Mab173 and DAXL-88 [106]. However, most of these drugs are still in preclinical trial stage and there is no available data regarding the efficacy [106]. Anti-AXL chimeric antigen receptor (CAR)-T-cell therapy is a new precise targeted immunotherapy for cancer, which is currently under clinical trials (NCT05128786 and NCT03393936). Due to its significant effect on AXL-positive osteomyeloid leukemia cells and TNBC cell models [107, 108], it could be a promising regime for cancer treatment. Another strategy targeting AXL is nucleic acid aptamers, a short stretch of nucleotide that can bind to specific target molecules with high affinity, low toxicity and are easy to synthesize [109]. The aptamer GL21.T fulfils these criteria, preliminary data show it binds AXL with high affinity, blocks AXL-dependent signal transduction pathway, and inhibits tumor migration and invasion [110]. However, more evidence is needed to support the application of these drugs in cancer treatment.

## Discussion and future perspectives

Drug resistance remains a significant challenge in cancer treatment, but recent studies suggest that AXL may be a promising target to address this issue. In addition to GAS6-dependent activation, AXL can be activated by interacting with various partners, such as PROS1 and EGFR, which are overexpressed in many cancers, particularly those that are drug-resistant. Because AXL plays a role in cancer resistance through multiple mechanisms, AXL-target drugs could be an effective strategy to alleviate drug resistance by inhibiting EMT transformation, interfering with DNA damage and DDR, inhibiting anti-tumor immune microenvironment, attenuating reactivation of oncogenic bypass, metabolic disorder and so on. Numerous ongoing clinical trials are targeting AXL alone or in combination with other drugs, demonstrating significant clinical therapeutic effect. However, several issues need to be considered when using combination therapy with AXL inhibitors. AXL activity is potent and extensive in many biological processes, and the side effects resulting from AXL targeting treatment should be carefully evaluated in clinical application. Since combination therapy does not always generate curative effect, it may inhibit immunity and metabolic remodeling at some point, and this needs to be further clarified to provide clear guidance for combined immunosuppressive therapy.

With the development of nanomedicine, the drug delivery system based on nanocarriers (NDDS) shows a good application prospect in cancer treatment [111]. NDDS can not only improve the solubility of chemotherapy drugs and deliver higher doses of drugs, but also reduce the toxicity of systemic chemotherapy to normal tissues [112, 113]. Based on this, the use of nanocarriers to deliver AXL inhibitors may be an effective strategy to reduce their toxic side effects and enhance their efficacy. However, there are still many challenges that need to be addressed before nanotechnology can achieve widespread clinical application.

In short, targeting AXL is a promising new strategy to delay or even eliminate the development of drug resistance due to its extensive biological effects and functional diversity. As AXL-targeted drug improve and the underlying mechanism of AXL-drug resistance is better understood, AXL inhibition is expected to provide promising strategies for the treatment of cancer patients.

## Data Availability

Not applicable.

## Abbreviations

RTKs: Receptor tyrosine kinases
GAS6: Growth arrest-specific protein 6
NK: Natural killer
EGFR: Epidermal growth factor receptor
HER3: Human epidermal growth factor receptor 3
NSCLC: Non-small cell lung cancer
TWIST1: Twist family BHLH transcription factor 1
PLK1: Polo-like kinase 1
ccRCC: Clear cell renal cell carcinoma
HCC: Hepatocellular carcinoma
CCA: Cholangiocarcinoma
FNIII: Fibronectin type III
IG: Immunoglobulin
GLA: γ-Carboxyglutamic acid
LG: Laminin G
PROS1: Protein S
GBM: Glioblastoma
PDGFR: Platelet-derived growth factor receptor
MET: Mesenchymal to epithelial transition factor
EMT: Epithelial-mesenchymal transition
VEGF-A: Vascular endothelial growth factor-A
PI3K: Phosphoinositide 3-kinase
AKT: Protein kinase B
VEGFR-2: VEGF receptor-2
SFK: Src family kinase
TEAD: TEA domain
AP-1: Activation protein-1
HIF-1α: Hypoxia-inducible transcription factor-1α
JNK: C-Jun N-terminal kinase
miRNAs: Micro-RNAs
lncRNA: Long noncoding RNA
ST3GAL1: ST3 β-Galactoside α-2,3-Sialyltransferase 1
HSP90: Heat shock protein 90
CHIP: Carboxy terminus of HSP70 interacting protein
CBL-b: Casitas B lymphoma-b
ADAM: A disintegrin and metalloproteinases
sAXL: Soluble AXL
OS: Overall survival
PDAC: Pancreatic ductal adenocarcinoma
TKI: Tyrosine kinase inhibitors
SCLC: Small-cell lung cancer cells
ESCC: Esophageal squamous cell carcinoma
OSCC: Oral squamous cell carcinoma
GSK-3β: Glycogen synthase kinase 3β
ZEB1: Zinc finger E-box binding homeobox 1
TGF-β: Transforming growth factor-β
SMAD3: SMAD family member 3
DDR: DNA damage response
RS: Replication stress
ROS: Reactive oxygen species
DSB: Double-strand breaks
HR: Homologous recombination
TLS: Translesion synthesis
PCNA: Proliferating cell nuclear antigen
CHK1: Checkpoint kinase 1
CDC2: Cyclin dependent kinase 1
ERK: Extracellular signal-regulated kinase
p90RSK: 90 KDa ribosomal S6 kinase
WEE1: WEE1 G2 checkpoint kinase
PD-L1: Programmed cell death ligand 1
ICAM1: Intercellular adhesion molecule 1
ULBP1: UL16 binding protein 1
CTL: Cytotoxic T lymphocytes
LFA-1: Lymphocyte function-associated antigen 1
STK11/LKB1: Serine/threonine kinase 11
TBK1: TANK binding kinase 1
NF-κB: Nuclear factor-κB
MAPK: Mitogen-activated protein kinase
PKM2: M2 isoform of pyruvate kinase
NRG1: Neuromodulator 1
CAR: Chimeric antigen receptor
MZF-1: Myeloid zinc finger 1
EZH2: Enhancer of zeste homolog 2
NFI: Nuclear factor I
SP1/SP3: Specificity protein 1/3
hnRNP-L: Heterogeneous nuclear ribonucleoprotein-L
ZNF224: Zinc finger protein 224
PTBP1: Polypyrimidine tract binding protein 1
METTL3: Methyltransferase 3
JAK: Janus kinase, STAT1: signal transducer and activator of transcription 1
SOCS1: Suppressor of cytokine signaling 1
mTOR: Mechanistic target of rapamycin kinase
Ang-2: Angiogenin-2
